# Real-world outcomes of lenvatinib plus pembrolizumab in intermediate- and poor-risk metastatic renal cell carcinoma

**DOI:** 10.37349/etat.2025.1002305

**Published:** 2025-04-01

**Authors:** Ilya Tsimafeyeu, Alexander Sultanbaev, Daria Dubovichenko, Makhabbat Murzalina, Alexander Volkov, Rashida Orlova, Igor Utyashev, Georgy Malina, Mark Gluzman

**Affiliations:** IRCCS Istituto Romagnolo per lo Studio dei Tumori (IRST) “Dino Amadori”, Italy; ^1^Bureau for Cancer Research (BUCARE) – Moscow office, 109147 Moscow, Russian Federation; ^2^Republican Clinical Oncology Dispensary, 450054 Ufa, Russian Federation; ^3^Arkhangelsk Regional Cancer Center, 163045 Arkhangelsk, Russian Federation; ^4^Orenburg Regional Cancer Center, 460021 Orenburg, Russian Federation; ^5^PET Technology, Nuclear Medical Сenter, 450075 Ufa, Russian Federation; ^6^Saint-Petersburg State University, 199034 Saint-Petersburg, Russian Federation; ^7^Hadassah Medical Moscow, 143026 Moscow, Russian Federation; ^8^Moscow Oncology Hospital No. 62, 143515 Moscow, Russian Federation

**Keywords:** Metastatic renal cell carcinoma, lenvatinib plus pembrolizumab, real-world evidence, IMDC intermediate and poor risk

## Abstract

The combination of lenvatinib and pembrolizumab (Len + Pembro) demonstrated significant efficacy in the phase 3 CLEAR study for metastatic renal cell carcinoma (RCC). However, poor-risk patients represented only a small proportion of the trial population. This multicenter retrospective cohort study assessed the real-world efficacy and safety of Len + Pembro in patients with clear-cell metastatic RCC and intermediate or poor International Metastatic RCC Database Consortium risk. Outcomes included objective response rate (ORR), progression-free survival (PFS), overall survival (OS), and safety. Sixty patients were analyzed, with a median age of 56 years. Poor risk was identified in 53% of patients, and 90% had metastases to ≥ 2 organs. ORR was 48.33%, disease control rate was 86.7%, and median PFS was 19.0 months. Grade ≥ 3 adverse events occurred in 25% of patients, with 33.3% requiring lenvatinib dose reductions. Lenvatinib plus pembrolizumab demonstrated robust efficacy and a manageable safety profile in a real-world population with advanced disease and poor-risk features, consistent with outcomes reported in clinical trials.

## Introduction

Renal cell carcinoma (RCC) accounts for 90% of all kidney cancers, with clear-cell RCC being the most common histological subtype [1]. Approximately 60% of patients present with advanced disease or develop metastases after surgery and require systemic therapy [2]. Advances in immunotherapy and targeted agents have transformed the treatment landscape for metastatic clear-cell RCC [3].

The combination of lenvatinib, a multikinase inhibitor, and pembrolizumab, a programmed cell death protein 1 (PD-1) immune checkpoint inhibitor, showed significant improvements in survival and response rates compared to sunitinib in the phase 3 CLEAR study [4]. Despite the encouraging results, poor-risk patients, defined by the International Metastatic RCC Database Consortium (IMDC) criteria, comprised only 9.3% of the CLEAR study population [5]. Real-world evidence evaluating lenvatinib plus pembrolizumab in poor and intermediate-risk patients is limited. This study aims to fill this gap by assessing the efficacy and safety of lenvatinib plus pembrolizumab in a routine clinical setting.

## Materials and methods

This multicenter retrospective cohort study included patients with metastatic clear-cell RCC treated with lenvatinib plus pembrolizumab as first-line therapy between 2019 and 2023. Eligible patients were ≥ 18 years old, had poor or intermediate IMDC risk, and had measurable disease per Response Evaluation Criteria in Solid Tumors (RECIST, version 1.1). Patients with prior systemic therapy, untreated central nervous system (CNS) metastases and uncontrolled medical conditions (such as unstable angina pectoris, recent myocardial infarction, symptomatic congestive heart failure, acquired or inherited bleeding disorders, or thrombosis) were ineligible for this trial.

Eligible patients received oral lenvatinib (20 mg daily) in combination with intravenous pembrolizumab (200 mg every three weeks or 400 mg every six weeks) until disease progression or unacceptable toxicity. Dose adjustments were permitted for lenvatinib based on toxicity.

The primary endpoint was objective response rate (ORR), defined as the proportion of patients achieving complete or partial responses. Secondary endpoints included progression-free survival (PFS), overall survival (OS), disease control rate (DCR; defined as complete response, partial response, or stable disease), and safety, including treatment-related adverse events (TRAEs) according to Common Terminology Criteria for Adverse Events (version 5.0).

Baseline characteristics and outcomes (ORR, TRAEs rate) were summarized using descriptive statistics. Kaplan-Meier analysis was used to estimate PFS and OS, with 95% confidence intervals (CI). Follow-up time was defined as the interval between the start of first-line therapy and the last patient follow-up (PFS) or death (OS).

This study adhered to the principles outlined in the Declaration of Helsinki and received approval from the Principal Investigators and Study Group (approval number: KCRB12012023-01). All patients provided written informed consent to receive the immunotargeted therapy.

## Results

A total of 60 patients were included, with a median age of 56 years (range, 36–73); 70% were male. Poor-risk IMDC criteria were met by 53% of patients, while 47% had intermediate risk. The majority (90%) presented with ≥ 2 metastatic sites, including liver (33%) and bone (15%) metastases. Sarcomatoid features were observed in 18.3% of tumors. Table 1 summarizes patient and tumor characteristics.

**Table 1:** Patient characteristics

**Characteristics**	**Patient**
***N* (%)**	60 (100)
**Age, median, years (range)**	56 (36–73)
**Sarcomatoid RCC, *N* (%)**	11 (18.3)
**Organs with metastases, *N* (%)**	
1 ≥ 2	6 (10) 54 (90)
**Sites of metastases, *N* (%)**	
lungs lymph nodes liver bones soft tissue adrenal brain	40 (67) 40 (67) 20 (33) 9 (15) 8 (13) 7 (12) 2 (3)
**Tumor thrombus, *N* (%)**	4 (7)
**ECOG PS, *N* (%)**	
0 1 2 3	6 (10) 39 (65) 12 (20) 3 (5)
**History of nephrectomy, *N* (%)**	
Yes No	52 (87) 8 (13)
**IMDC risk, *N* (%)**	
Intermediate Poor	28 (47) 32 (53)

RCC: renal cell carcinoma; ECOG PS: Eastern Cooperative Oncology Group Performance Status; IMDC: International Metastatic RCC Database Consortium

**Figure 1:**
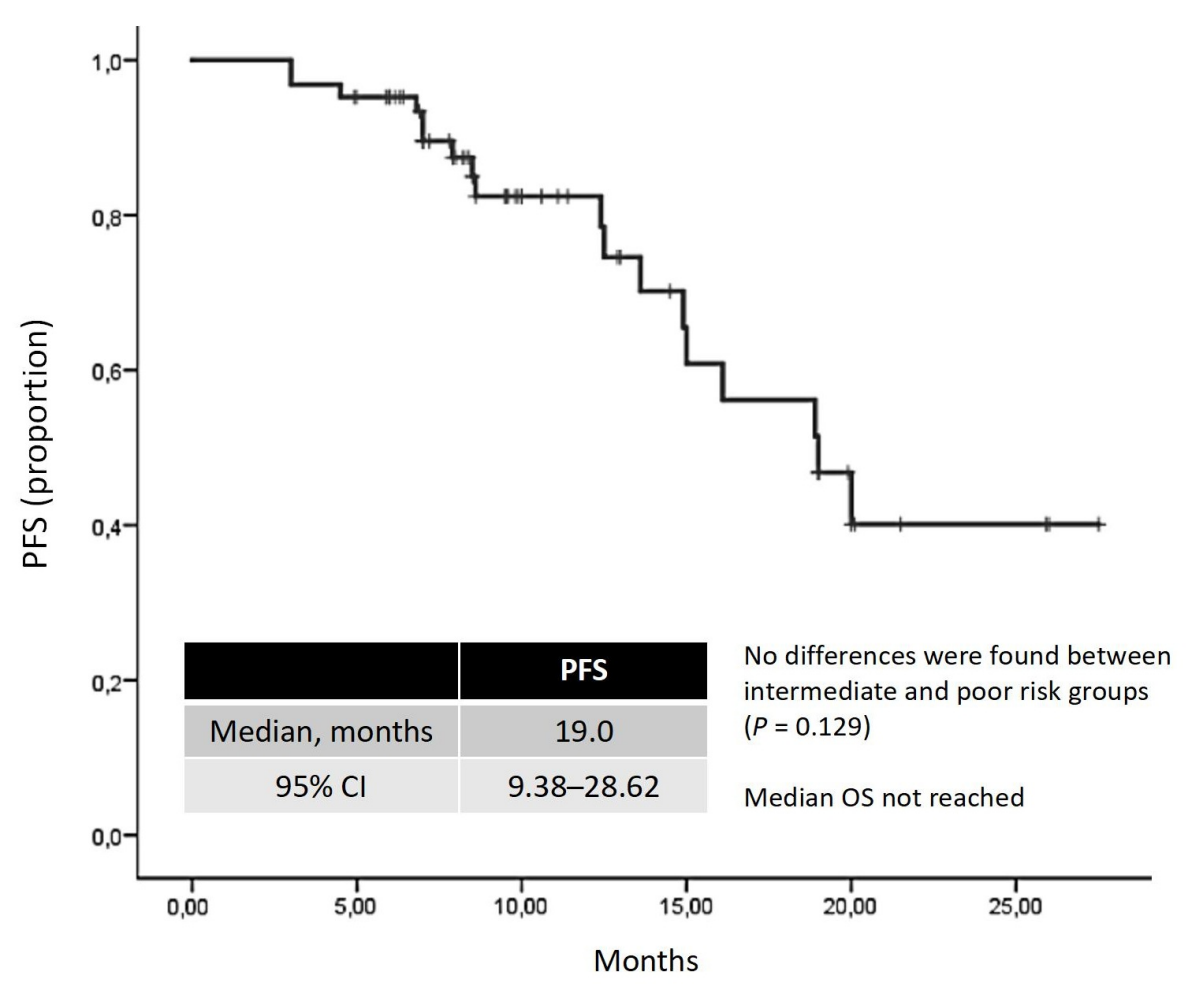
**Kaplan-Meier curve for PFS.** PFS: progression-free survival; CI: confidence intervals; OS: overall survival

The median duration of lenvatinib plus pembrolizumab therapy was 15.7 months. The ORR was 48.33%, with 5.0% achieving complete responses and 43.33% achieving partial responses. Stable disease was observed in 38.33%, resulting in a DCR of 86.7% (Table 2). At the median follow-up time of 20.4 months, the median PFS was 19.0 months (95% CI, 9.38–28.62; Figure 1). The median OS was not reached at the time of analysis.

**Table 2:** Real-world antitumor activity of lenvatinib and pembrolizumab

**Variable**	***N* (%)**
Complete response	3 (5.0)
Partial response	26 (43.33)
ORR	29 (48.33)
Stable disease	23 (38.33)
Disease control rate	52 (86.7)
Progressive disease	8 (13.3)

ORR: objective response rate

Grade ≥ 3 TRAEs occurred in 15 patients (25%), with the most common events being hypertension (13.3%), fatigue (8.3%), and diarrhea (8.3%); Table 3. Lenvatinib dose reductions were required in 20 (33.3%) of patients, and 3 (5%) discontinued lenvatinib due to toxicity. Pembrolizumab discontinuation was not reported.

**Table 3:** Grade ≥ 3 adverse events

**TRAEs**	***N* (%)**
All grade ≥ 3 adverse events	15 (25.0)
Fatigue	5 (8.3)
Diarrhea	5 (8.3)
Hypothyroidism	3 (5.0)
Proteinuria	2 (3.3)
Decreased appetite	1 (1.7)
Nausea	1 (1.7)
Anemia	1 (1.7)
Stomatitis	1 (1.7)

TRAEs: treatment-related adverse events

## Discussion

This real-world study demonstrates the robust efficacy and manageable safety of lenvatinib plus pembrolizumab in intermediate- and poor-risk metastatic clear-cell RCC patients, complementing findings from the CLEAR trial.

The ORR of 48.33% observed in this study was lower compared to the 71.3% reported in the CLEAR study [6]. This discrepancy may be attributed to differences in patient populations, as our cohort included a higher proportion of poor-risk patients (53% vs. 9.3%), a greater burden of metastatic disease (≥ 2 metastatic organs in 90% vs. 71.3%), and higher rates of sarcomatoid features (18.3% vs. 7.9%). These factors are known to negatively impact outcomes, emphasizing the complexity of treating real-world metastatic clear-cell RCC populations. The first study evaluating real-world outcomes of lenvatinib plus pembrolizumab in treatment-naïve metastatic clear-cell RCC reported a higher ORR of 66% [7]. This elevated number may be attributed to the inclusion of patients with favorable IMDC risk, comprising 14% of the 50-patient cohort.

The median PFS of 19.0 months is similarly moderately reduced compared to the 23.9 months reported in the CLEAR trial. The presence of liver metastases in one-third of our cohort may have contributed to this difference, given the historically poor prognosis associated with hepatic involvement in metastatic clear-cell RCC. Other prognostically unfavorable metastatic sites, such as bones, brain, adrenal glands, and tumor thrombus, may also impact the PFS.

Importantly, the safety profile of lenvatinib plus pembrolizumab in this study aligns with prospective trial data. The incidence of grade ≥ 3 TRAEs (25%) was lower than the 84.9% observed in the CLEAR trial, potentially reflecting more conservative dose adjustments in routine practice. Lenvatinib dose reductions, implemented in one-third of patients, effectively mitigated toxicity without compromising efficacy, underscoring the importance of individualized treatment adjustments. In an earlier Japanese study [7], adverse events occurred in all patients (100%), with grade ≥ 3 events reported in 66% of cases. The reduction in serious adverse events observed in our study may be attributed to improved experience with immunotargeted therapy and the proactive management of TRAEs at earlier stages.

Lenvatinib plus pembrolizumab remains a compelling option for intermediate- and poor-risk metastatic clear-cell RCC patients. Its combined mechanism of action, integrating vascular endothelial growth factor (VEGF)/fibroblast growth factor receptor (FGFR) inhibition with immune checkpoint blockade [8, 9], offers broad efficacy across various disease presentations, including cases with sarcomatoid differentiation and multiple metastatic sites [10].

Nevertheless, this study has several limitations. First, it is retrospective design and reliance on real-world data may introduce selection and reporting biases, as treatment practices and documentation standards can vary across centers. Second, the relatively small sample size limits the generalizability of the findings and reduces the statistical power for subgroup analyses. Third, the absence of a direct comparator arm makes it difficult to draw definitive conclusions about the relative efficacy and safety of this regimen compared to other first-line therapies in similar patient populations. Prospective studies with larger cohorts and biomarker integration are warranted to validate these findings.

In conclusion, our real-world study confirms the efficacy and safety of lenvatinib plus pembrolizumab in intermediate- and poor-risk metastatic clear-cell RCC patients. The ORR, PFS, and manageable toxicity observed in this cohort validate its role as a first-line therapy, particularly in patients with advanced disease and unfavorable prognostic features. Future research should also focus on identifying predictive biomarkers and optimizing subsequent treatment strategies to further improve outcomes for this challenging population.

## Abbreviations

CI: confidence intervals
DCR: disease control rate
IMDC: International Metastatic Renal Cell Carcinoma Database Consortium
ORR: objective response rate
OS: overall survival
PFS: progression-free survival
RCC: renal cell carcinoma
TRAEs: treatment-related adverse events

## Declarations

### Availability of data and materials

The raw data are not available due to confidentiality.

### Copyright

© The Author(s) 2025.
